# Clinical Decision Making of Nurses Working in Hospital Settings

**DOI:** 10.1155/2011/524918

**Published:** 2011-09-28

**Authors:** Ida Torunn Bjørk, Glenys A. Hamilton

**Affiliations:** ^1^Department of Nursing Science, University of Oslo, Postboks 1153, Blindern, 0318 Oslo, Norway; ^2^Research Department, GAH Consulting, 601 George Hill Road, Lancaster, MA 01523, USA

## Abstract

This study analyzed nurses' perceptions of clinical decision making (CDM) in their clinical practice and compared differences in decision making related to nurse demographic and contextual variables. A cross-sectional survey was carried out with 2095 nurses in four hospitals in Norway. A 24-item Nursing Decision Making Instrument based on cognitive continuum theory was used to explore how nurses perceived their CDM when meeting an elective patient for the first time. Data were analyzed with descriptive frequencies, *t*-tests, Chi-Square test, and linear regression. Nurses' decision making was categorized into analytic-systematic, intuitive-interpretive, and quasi-rational models of CDM. Most nurses reported the use of quasi-rational models during CDM thereby supporting the tenet that cognition most often includes properties of both analysis and intuition. Increased use of intuitive-interpretive models of CDM was associated with years in present job, further education, male gender, higher age, and working in predominantly surgical units.

## 1. Introduction

In the clinical setting, nurses are continually faced with demands to make decisions of care. The process of coming to a choice is the essence of decision making. This process is viewed as complex [1, 2]. O'Neill et al. [3] suggest that the complexity of clinical decision making (CDM) requires a broad knowledge base and access to reliable sources of information, as well as working in a supportive environment. The decisions nurses make while performing nursing care will influence their effectiveness in clinical practice and make an impact on patients' lives and experiences with health care regardless of which setting or country the nurse is practicing in. Knowledge about nurses' decision making is therefore of utmost importance. Understanding how nurses make decisions is also a prerequisite to facilitating learning and development of decision making skills in nursing education [1]. 

## 2. Background

Historically, CDM in nursing has been discussed in light of systematic-positivist models and the intuitive-humanist model [4]. Two approaches dominate in nursing research within the systematic-positivist stance, analytical decision making theory, and information-processing theory. Analytical decision making theory assumes that rational analytical thinking precedes action. The analysis is a systematic step-by-step procedure with the use of logical rules that can be followed until a decision is made [5]. The information-processing model is a psychological theory much used in research in medical decision making and characterized by a scientific approach to making decisions [6]. It is also termed the hypothetico-deductive approach [1, 4]. Hamers et al. [7] described four major stages of this process in nursing as, gathering preliminary clinical information about the patient, generating tentative hypotheses about the patients' condition, interpreting the initially registered cues in light of the tentative hypotheses, and weighing the decision alternatives before choosing the one that fits best in light of the evidence collected. Earlier knowledge acquired about the situation at hand is included in this process [8]. The intuitive-humanist model is best known in nursing through Benner's work [9]. Intuition has been defined in several ways, for example, “understanding without a rationale” [10, p.23] or “a perception of possibilities, meanings and relationships by way of insight” [11, p.63]. According to Benner [9], intuition is rooted in the ability to recognize patterns of cues. This is an ability that develops with experience in managing patients in the nursing field. According to Thompson [4, p.1224], the basic idea of the intuitive-humanist model is that, “intuitive judgment distinguishes the expert from the novice, with the expert no longer relying on analytical principles to connect their understanding of the situation to appropriate action.” The analytical and intuitive stance towards decision making have ardent followers and have often been viewed as two distinct types of cognitive activity sharply separated. However, since the late 1990s, a third approach to decision making has been discussed in the nursing literature, decision making based on the cognitive continuum theory (CCT) by Hammond [5].

 Hammond [5] does not view analysis and intuition as distinct cognitive systems. He offers instead the idea of a cognitive continuum where analysis and intuition are located at each end point. Cognition often falls between the end points and thereby includes properties of both analysis and intuition, referred to as quasi-rational cognition, meaning that many judgment tasks present cues that induce an oscillation between analytical and intuitive cognition [5]. A major tenet of the theory is that “judgment is a joint function of task properties and cognitive properties” [5, p.83], that is, different judgment tasks should be solved through different cognitive processes. In his theory, he therefore describes differences among judgment tasks and locates them in relation to cognitive properties along the cognitive continuum. A judgment task that involves uncertainty is difficult to break down into distinct components and may benefit from a more intuitive approach than a judgment task that is well structured with few and recognizable cues. The latter judgment task would favor a more analytical approach. Dowding [12] in a commentary on Banning's article [1] seems to support Hammond's [5] idea of viewing decision making within one theoretical system. She suggests that hypothetico-deductive reasoning, intuition, pattern matching, heuristics, and so forth. All lie within the psychological theory of information-processing theory.

The CCT has been tested in nonnursing settings [13–15]. Since 1999, several authors have suggested that Hammond's CCT could be a possible alternative way of conceptualizing decision making in nursing [4, 16, 17]. In two qualitative studies, CCT was used either as an explicit analytical tool [18] or as a theoretical perspective in the framing and discussion of the study [19]. Both studies concerned nurses' decision making during pharmacological management. Both studies also report a mismatch between the type of decision making nurses used and the characteristics of the situation, for example, that intuitive approaches were used when more analytic approaches should have been used [18], or that appropriate decision tools were missing to help nurses during their analytical approaches [19]. 

 In collaboration with international colleagues, Lauri and Salanterä [2] included Hammond's CCT [5] in a theoretical framework for developing an instrument to explore nurses' perception of their decision making at a general level, that is, the way in which nurses perceived to arrive at their decisions in practical nursing situations. The main purpose was to examine cognitive processes nurses thought they used in their decision making and correlate them with demographic and contextual factors. Based on both CCT and information processing theory, an extensive literature review, interviews with nurses, and former studies of decision making, a 56-item instrument was developed. According to the their content, the items in the questionnaire were organized to reflect four stages of CDM: (a) data collection, (b) data processing and identification of problems, (c) plans of action, and (d) implementation of plan, monitoring, and evaluation [2]. The instrument was used in a study with 1460 nurses from seven countries after testing.

Their study showed that nurses' use of CDM differed according to field of practice and country [20–23]. In relation to different stages of the decision making process, Lauri and Salanterä [2] claimed that pure intuitive decision making did not weight on any of the 4 stages. Analytical decision making did weight in stage 2, that is, data processing and identification of problems. The other stages of decision making were more or less quasi-rational. The authors were cautious in drawing any general conclusions about factors underlying the differences in nurses' perception of their decision making but suggested that it was fair to assume that “the instrument allows us to determine in general terms how nurses' decision making occurs on the continuum from analytical to intuitive” [2, p.98]. 

The present study was part of a larger study that had an overall purpose of exploring learning and professional development in hospital nurses. Inspired by the work of Lauri and Salanterä [2], the study aimed at exploring nurses' perception of their clinical decision making (CDM) in a specified patient situation. The following research questions were formulated.

1. What CDM models characterize the total sample of nurses?
2. What is the association between selected independent variables (background and demographic variables) and the dependent variable (CDM)?
3. How much variance in CDM can be explained by scores on the independent variables?
4. Is there any difference in CDM models across the four stages of the decision making process?

## 3. Methods

### 3.1. Design, Sample, and Setting

The study used a descriptive cross-sectional survey design in which nurses completed a questionnaire on one occasion. A convenience sample of registered nurses in clinical positions at four hospitals in the western and southern part of Norway was recruited. Two of the hospitals were affiliated with universities, the third was regional, and the fourth a local hospital. The inclusion criteria were nurses employed in clinical positions working half, or more than a half, of a full-time equivalent. Respondents received an envelope at work including a cover letter explaining the purpose of the study and ensuring confidentiality, the questionnaire, and a preaddressed envelope for return of their response. A total of 4,650 nurses were invited to participate in the study. A return rate of 45.5% yielded 2,095 questionnaires. The data were collected in 2004-5.

### 3.2. Instrumentation

The questionnaire included (1) demographic and background variables, (2) the 24-item Nursing Decision Making Instrument (Salanterä, e-mail correspondence 2004-5), (3) the index of work satisfaction [24], and (4) author-designed evaluative questions for participants in long-term in-house educational programs (clinical ladder programs). Results from the two latter sections are reported elsewhere [25, 26]. 

**Figure 1:**
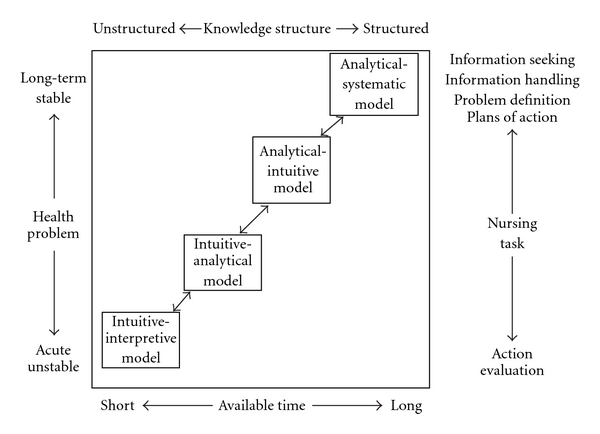
Nursing decision making theory based on Hammond's model of cognitive continuum theory (1996, p.235) (Salanterä, e-mail correspondence 2004-5).

 The 24-item Nursing Decision Making Instrument is a shortened version of the original 56-item instrument presented in the background of this article.  Figure 1 shows how Lauri and Salenterä related four decision making models to Hammond's CCT [5].

These four models are inserted in a continuum from analysis to intuition and defined by aspects of the patient's health problem, knowledge structure, nursing task, and available time, corresponding to Hammond's [5, p.235] concepts of task features and cognitive structures. Based on the international sample, Lauri and Salenterä [2] developed a scoring system to allow for assessment of nurses' decision making style. The scores were related to the decision making models presented in Figure 1: the intuitive-interpretive model, the intuitive-analytical model and the analytical-intuitive model constituting quasi-rational models of cognition, and the analytical-systematic model.

In E-mail correspondence with Salanterä (2004-2005), we were offered to use the 24-item Nursing Decision Making Instrument (Sanna Salanterä, Professor of Clinical Nursing Science, Department of Nursing Science, University of Turku, 20014 Turun Yliopisto, Finland, E-mail: sansala@utu.fi). For the shortened version of the instrument, cut-off points in the scores relating to the four decision making models were defined on the basis of quartiles: 25% of the responses were intuitive-interpretive, 25% were analytical-systematic, and 50% in the two middle quartiles were analytical-intuitive or intuitive-analytical, that is, quasi-rational [Salanterä, E-mail correspondence]. Equivalent to the original instrument, the 24-item instrument had four subscales, each with six items, corresponding to the four stages of the decision making process. Even numbered items reflected decision making in unstable tasks or situations with short available time, for example, “I make assumptions about forthcoming nursing problems during the first contact with the patient.” Odd numbered items reflected decision making in structured tasks or situations with enough time to seek or handle information or plan actions, for example, “On the basis of my advance iformation, I specify all the items I intend to monitor and ask the patient about.”

Respondents answered each question on a 5-point Likert-type scale with response options of “almost never,” “rarely,” “sometimes,” “often,” and “almost always.” These items were scored from 1 to 5 so the lowest scores measured analytical decision making and the highest scores intuitive decision making. On the even numbered items, the response option of “almost always” would then indicate a highly intuitive approach. The scores for responses to odd items were reversed; thereby, the response option of “almost never” would be scored as 5 and also indicate a highly intuitive approach. A low total score described analytical decision making and a high-score intuitive decision making. The scores were added up, and the sum total was interpreted following instructions from Salanterä [E-mail correspondence 2004-5]: 24–67 indicate analytical-systematic decision making, 68–77 indicate quasi-rational decision making, and 78–120 indicate intuitive-interpretive decision making. 

The respondents were instructed to answer the questionnaire with an elective patient in mind. An elective patient situation implies certain judgment tasks that differ from acute situations, that is, the difference in time at hand for colleting data about the patient, or discussing with colleagues the appropriateness of interventions. The idea was to set a scene that would prompt nurses to think of their decision making with the same type of judgment tasks in mind and thereby allow for comparison across hospitals and units. 

### 3.3. Ethical Considerations

According to Norwegian law, permission was not needed from the regional Committees for Medical and Health Research Ethics. Permission to use name lists in order to supply enough questionnaires to each unit was granted by the Director of Nursing or Director of Research according to local regulations, and such lists were obtained from the personnel department of each hospital. Permission to distribute questionnaires was obtained from department directors. Questionnaires could be related to each hospital but were otherwise anonymous. Informed consent to participate was indicated by return of the questionnaire. 

### 3.4. Data Analysis

The questionnaires were optically scanned. In the original research, there was a lack of information on how the authors addressed missing data. In accordance with general statistical procedures, we addressed missing data in the present study in the following way. In relation to research questions 1–3, the analysis aimed at matching each nurse's total sum with cut-off points for different decision making models. Respondents with more than 40% of the items missing were therefore taken out of the data set (*n* = 75, 3.6% of the total sample). An inspection of the data revealed that missing responses were often due to missing responses to all questions on the last page of the questionnaire. Any missing items in other respondents data sets were substituted with the respondents own mean score. For question 4, the purpose was to look more closely at decision making models within each stage of the decision making process. Each stage has six items. Any stage with more than two out of six items missing was taken out of the analysis. Where respondents had one or two missing items, these were substituted with the mean score within that stage of decision making. These procedures resulted in a final *N* = 2020 for total score analysis and a variation in *N* of 2061, 2054, 1974, and 2009 for analysis within the four stages of CDM, respectively. Data were analyzed with frequency distributions, and inferential statistics. When studying the association between potentially predictive variables and a dependent variable, linear regression analysis can be used. Multicollinearity was controlled by the coefficients tolerance (>0.5), and variance inflation factor (VIF, close to 1 < 2) Cook's D and Mahalanobis D, and standard residuals were used to identify possible outliers that might distort the statistics [27, p.128]. Adjusted R square evaluated the variance that the independent variables contributed to explaining the association with the dependent variable, CDM. The statistical package for social sciences version 15.0 was used for statistical analysis.

### 3.5. Reliability and Validity

Authors of the original 56-item instrument [2] developed the shortened version thru a factor analysis on the original instrument to ensure construct validity, reduction of items by keeping items that had a high impact in the factor analysis, and reformulation of items according to responses in the previous measurements. The shortened version of the instrument has not been formally validated. The questionnaire was translated back-and-forth from English to Norwegian. A Norwegian person fluent in English translated from English to Norwegian, an English person translated the Norwegian version back to English, and finally this version was compared with the original. Only a few small corrections were necessary. Cronbachs alpha in the present study was 0.863. A manual check was performed of questionnaires where data cleaning procedures uncovered abnormal values.

## 4. Findings

The study participants ranged in age from 21 to 68 (mean 37.5 years), 7.9% were men, 8.3% of the nurses had worked more than 5 years in their unit, 66% had graduated before 1999, and average work experience in their present setting was 4.9 years. Approximately 40% of the participants had formal continuing education between 1/2 and 1.5 years, and 25.8% of the nurses had completed or were enrolled in a 5-year in-house clinical ladder program.

### 4.1. What CDM Models Characterize the Total Sample of Nurses?

**Figure 2:**
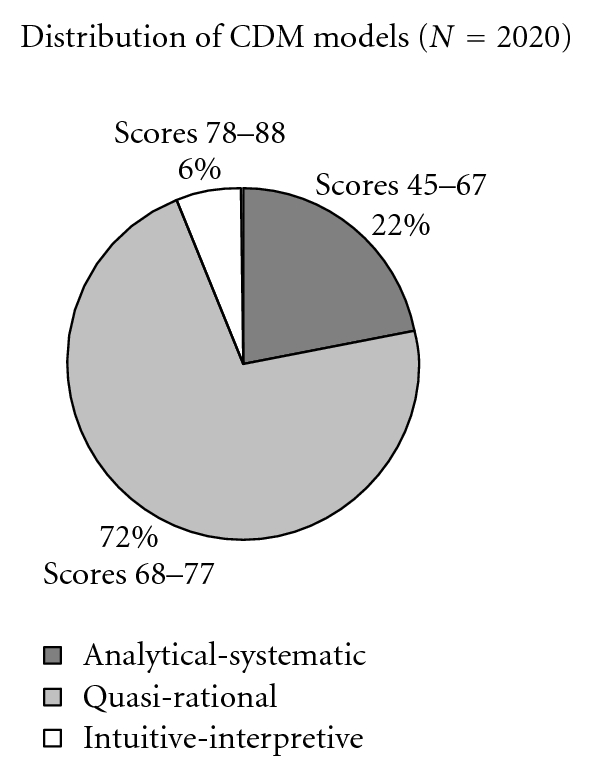
Distribution of CDM models among the total sample of nurses.

The possible range of scores in the CDM instrument was 24–120. In the whole sample, the range in scores was 45–88 (mean 70.65, SD 4.35). The distribution of CDM models as reported by the total sample of nurses is shown in the chart in Figure 2. 

Figure 2 indicates that most nurses reported the use of quasi-rational models during CDM. Few nurses fell within the score boundaries indicating the use of intuitive-interpretive models. 

### 4.2. What is the Association between Selected Independent Variables (Background and Demographic Variables) and the Dependent Variable (CDM)?

Background and demographic variables were computed with *t*-tests for nominal data and Pearson's *r* for interval level data. The variables that were statistically associated with CDM are reported in Table 1.

### 4.3. How Much Variance in CDM Can Be Explained by Scores on the Independent Variables?

Variables statistically associated with CDM were entered into the final regression. ANOVA statistics are reported in Table 2. 

Table 2 shows that nurses' number of years in present job and further education had the largest associations with CDM.

### 4.4. Is There Any Difference in CDM Models within the Four Subscales of the CDM Instrument?

**Figure 3:**
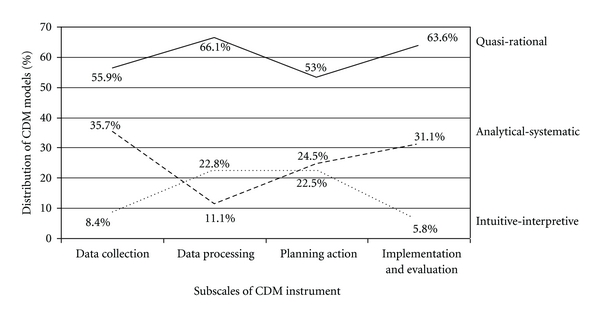
Pattern of reported CDM models within the subscales of the CDM Instrument.

Differences in nurses' reported use of CDM models across the four subscales of the CDM instrument are illustrated in Figure 3.

In general, nurses reported the use of quasi-rational models of CDM more often than either analytical-systematic or intuitive-interpretive models. However, the largest variations across the stages of decision making occurred in relation to reported use of the two latter models.

Figure 3 shows that the percentage of nurses reporting the use of analytical-systematic models was highest during data collection and implementation and evaluation. Correspondingly, interpretive-intuitive models were low in use during these stages, higher in use during data processing and used approximately as much as the analytical-systematic model during the stage of planning action.

 There were variations in the pattern illustrated in Figure 3 when demographic and contextual variables were taken into account. These variations are illustrated in Table 3. 

It is clear that participation in clinical ladders had no significant impact on nurses' reported use of CDM models across any of the stages of the decision making process. Age and nurses' field of practice was also variables that had little impact across the four subscales. 

## 5. Discussion

The purpose of this study was to explore the cognitive processes used during CDM as reported by a large sample of Norwegian nurses and to identify how demographic and contextual variables were associated with decision making. Since no studies with the shortened CDM instrument have yet been reported, direct comparisons are not possible although results from the present study may be compared with trends in Lauri and Salanterä's research [20, 22, 28]. Based on the scoring system developed for the shortened instrument, results from the present study show that in the whole sample of nurses the “window” for perceived quasi-rational approaches in CDM is large. This is similar to nurses in Dowding et al.'s study [19] and with Hammond's [5] suggestion that it is most common to oscillate between analytical and intuitive modes of cognition during decision making. Also, the analytical-systematic model is perceived to be much more in use than the intuitive-interpretive model. One interpretation of this may relate to the character of the task outlined in the questionnaire. An elective patient situation affords a reasonable amount of time for decision making and is relatively well-structured. This situation therefore has properties that may induce analysis [16, 29]. 

 Since the CDM instrument has a lower number for analytical-systematic CDM and a higher for intuitive- interpretive, with quasi-rational decision making modes in between, one can conclude that years in present job is significantly associated with intuitive-interpretive CDM, followed by further education, male gender, higher age, and surgical field of practice. In line with findings in Benner and colleagues' research [9, 30, 31], there is a significant increase in the nurses' reported use of intuitive-interpretive CDM models with increasing experience in their unit. This is also similar to Lauri et al.'s [20] report on CDM among nurses in geriatric and acute care settings in Finland, Sweden, Switzerland, Canada, and the USA. In other studies, however, experience does not significantly influence CDM [22, 28]. Further education was also associated with perceptions of more intuitive decision making among Norwegian nurses. Although earlier research into the association between educational level and decision making is inconclusive [32], Lauri et al., [20] found that nurses with professional education used significantly more intuitive CDM than nurses with only 2.5–3 years of education. As age is also a significant factor associated with CDM models in the present study, and both further education and years of work experience often parallel increasing age, it is difficult to gauge the contributions of these demographic variables. 

 An interesting finding is the association between male gender and CDM. Male nurses' CDM scores are similar to that of nurses who had more than 10 years experience in their unit. However, male nurses had fewer years of experience less further education and were younger. This indicates that being male in itself may influence perceived models of CDM. Studies reporting on the association between gender and decision making are scarce. In the field of human relationships and management, Burke and Miller [33] found minimal support for a gender-based stereotype of women's intuition. From 51 interviews with seasoned professionals, they found that men were believed to use intuitive skills at work as much or more than women. In contrast, in a study of 520 physicians, nurses and health managers, men preferred rational reasoning while women preferred intuitive reasoning [34]. 

 Nurses' perception of their CDM in this study is associated with field of practice, as nurses in predominantly surgical units are more intuitive interpretive than nurses in predominantly medical units. Patients in surgical units may experience more sudden shifts in their health condition than patients in medical units. Nurses in a surgical field of practice may therefore be faced with tasks characterized by uncertainty and many cues at the same time. Such situations favor an intuitive approach in CDM [5]. 

 In the whole sample, variations in CDM models are also apparent across the different stages of the decision making process. As mentioned before, nurses in general use quasi-rational models of CDM the most. Analytical-systematic models of CDM are perceived to be more in use than intuitive-interpretive models during stages of data collection and implementation and evaluation. Intuitive-interpretive models are reported in use more during data processing, while during planning both models are perceived to be equally in use. This does not match the findings of Lauri and Salanterä [2], where one of their major findings was that analytical decision making models were weighted for the stage of data processing in all nursing fields. The nurses in the present study were prompted to relate their answers to how they viewed their CDM with an unknown but elective patient. To our knowledge, this was not done in the study by Lauri and Salanterä [2] and may be one reason for the difference in these findings. However, when CDM across stages of the decision making process is analyzed according to demographic variables, some groups of nurses do report more analytical-systematic models during data processing than other groups. 

### 5.1. Limitations

Although the sample in this study was large, a survey method has limitations as answers to a questionnaire may not represent nurses' actual decision making. Self-reported data may potentially bias the association being investigated. This is a limitation. However, we do not have any reason to believe that the questions were viewed as sensitive in any way. The respondents were also informed in the questionnaire that there were no right or wrong answers. Since this was a sample of convenience, it may not be representative of all nurses in Norway. We also acknowledge that this study was carried out in one country in Scandinavia and thus may not reflect CDM use in other countries. The analysis was also limited to an elective patient situation. There is a potential for nonresponse bias with a response rate of 45.5%. Personnel departments at Norwegian hospitals do not make lists of their employees that automatically include, for example, gender and age, so it is difficult to access such data to verify the demographic similarity between responders and nonresponders. However, all Norwegian RN's have the same undergraduate nursing education as there is only one form of educational program in the country. The limitation of mean imputation methods is discussed in the literature although mean substitution for items in multiple-item scales is often used [35] in order not to waste information by scoring the entire scale as missing. When data were inspected, the magnitude of missing was rather small and evenly distributed among the items (between 27–40 responses to the first 12 items, and between 61–65 responses to the last 12 items).

## 6. Conclusion

The research presented in this paper extends our ways of looking at CDM based on Hammond's [5] new insights into possible models of CDM. Our findings support the prevalence of nurses' oscillation between analysis and intuition, at least when nurses were confronted with the kind of decision situation introduced in this study. The exploratory nature of this work does not invite definitive conclusions about nurses' decision making. However, we believe it can stimulate ideas and discussions about additional ways of understanding the thinking processes nurses use in practice. This is the first time the shortened version of Lauri and Salanterä's [2] Nursing Decision Making Instrument is reported. More extensive evaluation of the CDM model in other countries and in different practice settings is therefore needed in order to explore the merit of this way of conceptualizing nurses' CDM. 

## Figures and Tables

**Table 1:** The association between background and demographic variables, and CDM.

	Pearsons *r*	*t*-test Mean	*P*
Age	0.059		0.01
Years in present job	0.132		0.01
Field of practice			
Predominantly surgical		71.04	
Predominantly medical		70.40	0.002
Further education			
Further education		71.22	
No further education		70.13	<0.000
Gender			
Male		71.62	
Female		70.56	0.003

**Table 2:** Amount of variance in CDM explained by independent variables.

Independent variables	Beta	*t*	*P*
Years in present job	0.142	5.33	<0.0001
Further education	0.126	4.97	<0.0001
Male gender	0.069	3.13	0.002
Higher age	0.081	2.77	0.006
Surgical field of practice	0.05	2.23	0.026

*F* = 15.698, *P* < 0.0001, *R*²  0.38.

**Table 3:** The influence of demographic and contextual variables on the use of analytical-systematic (A-S) and intuitive-interpretive (I-I) models within stages of the decision making process (Chi-Square, significance level *P* < 0.05).

Demographic and contextual variables	Data collection *P*	Data processing *P*	Planning action *P*	Implemen tation and evaluation *P*
Work in ward in years: <2, 2–4, 5–9, ≥10	Less A-S and more I-I as experience in ward increases 0.001	Less A-S and more I-I as experience in ward increases 0.007	Ns	Less A-S and more I-I as experience in ward increases 0.003
Further education: yes or no	Less A-S and more I-I if nurse has further education 0.002	More A-S if nurse has further education 0.039	Less A-S and more I-I if nurse has further education <0.001	Less A-S and more I-I if nurse has further education <0.001
Gender of nurse: male or female	Less A-S and more I-I if nurse is male .032	More A-S and less I-I if nurse is male .024	Less A-S and more I-I if nurse is male .001	Ns
Age in years: <37 or >37	Less A-S and more I-I if age over mean 0.015	Ns	Ns	Ns
Participation in Clinical ladder: yes or no	Ns	Ns	Ns	Ns
Type of hospital where nurses worked: local or regional, or university	Ns	No clear pattern 0.018	No clear pattern <0.001	Ns
Nurses' field of practice: predominantly surgical or medical	Ns	Less A-S and more I-I if nurses field of practice is predominantly surgical <0.001	Ns	Ns
